# New Data on the *Clevosaurus* (Sphenodontia: Clevosauridae) from the Upper Triassic of Southern Brazil

**DOI:** 10.1371/journal.pone.0137523

**Published:** 2015-09-10

**Authors:** Annie Schmaltz Hsiou, Marco Aurélio Gallo De França, Jorge Ferigolo

**Affiliations:** 1 Departamento de Biologia, Faculdade de Filosofia, Ciências e Letras de Ribeirão Preto, Universidade de São Paulo, Ribeirão Preto, São Paulo, Brazil; 2 Colegiado de Ciências Biológicas, Universidade Federal do Vale do São Francisco, Petrolina, Pernambuco, Brazil; 3 Seção de Paleontologia, Museu de Ciências Naturais, Fundação Zoobotânica do Rio Grande do Sul, Porto Alegre, RS, Brazil; New York Institute of Technology College of Osteopathic Medicine, UNITED STATES

## Abstract

The sphenodontian fossil record in South America is well known from Mesozoic and Paleogene deposits of Argentinean Patagonia, mainly represented by opisthodontians, or taxa closely related to the modern *Sphenodon*. In contrast, the Brazilian fossil record is restricted to the Caturrita Formation, Late Triassic of Rio Grande do Sul, represented by several specimens of Clevosauridae, including *Clevosaurus brasiliensis* Bonaparte and Sues, 2006. Traditionally, Clevosauridae includes several Late Triassic to Early Jurassic taxa, such as *Polysphenodon*, *Brachyrhinodon*, and *Clevosaurus*, the latter well-represented by several species. The detailed description of the specimen MCN-PV 2852 allowed the first systematic revision of most *Clevosaurus* species. Within Clevosauridae, *Polysphenodon* is the most basal taxon, and an IterPCR analysis revealed *Brachrhynodon* as a possible *Clevosaurus*; *C*. *petilus*, *C*. *wangi*, and *C*. *mcgilli* as possibly distinct taxonomic entities; and the South African *Clevosaurus* sp. is not closely related to *C*. *brasiliensis*. These data indicate the need of a deep phylogenetic review of Clevosauridae, in order to discover synapomorphic characters among the diversity of these Triassic/Jurassic sphenodontians.

## Introduction

The sphenodontian fossil record in South America is well-known from the Mesozoic deposits of Argentinean Patagonia [1–4]. These are mainly represented in the Late Cretaceous, with Cenomanian-Turonian eilenodontine opisthodontians and Late Campanian fragmentary remains most likely related to opisthodontians [5–9], in addition to the Late Campanian to Early Maastrichtian record of a partial tooth bearing maxilla that closely resembles those of modern *Sphenodon* [10]. More recently, *Sphenocondor gracilis* from the Middle Jurassic of Patagonia was described as the first South American basal rhynchocephalian [2] and the first South American Paleogene sphenodontian, *Kawasphenodon peligrensis*, was described for the early Paleocene of Patagonia [4]. For the early Mesozoic (Late Triassic) of South American, sphenodontian opisthodontians are known from northern Argentina [3] and clevosaurs from south Brazil [11,12]. Traditionally, clevosaurs comprise the Late Triassic *Polysphenodon* (Germany) and *Brachyrhinodon* (UK) [13–16] and the better-know genus *Clevosaurus*, represented by several Late Triassic–Early Jurassic species.

Late Triassic *Clevosaurus* record includes UK, Belgium, North America, China, and Brazil [11,17]; whereas Early Jurassic records of the genus includes North America, South Africa, UK, and Zimbabwe [17,18]. Currently there are six recognized *Clevosaurus* species: *C*. *hudsoni* (the type-species) [19,20], *C*. *bairdi* [21], *C*. *convalis* [18], *C*. *latidens* [22], *C*. *minor* [20], *C*. *brasiliensis* [11,12], whereas the Early Jurassic Chinese *Clevosaurus* from the Lufeng Formation (*C*. *mcgilli*, *C*. *petilus*, *and C*. *wangi*) described by Wu [23], were interpreted as *Clevosaurus* sp. [17]. It is noteworthy that, in a recent phylogenetic analysis, one specimen associated to *C*. *latidens* was considered a basal Opisthodontia, close to *Ankylosphenodon* from the Early Cretaceous of Mexico [24], forming the sister-group with all other opisthodontians, including *Sphenotitan* from the Late Triassic of Argentina [3].

The first sphenodontian formally described for the Late Triassic of Brazil was *Clevosaurus brasiliensis* Bonaparte and Sues, 2006 [11], with several specimens attributed to this taxon [12], including that shortly described by Ferigolo [25–28]. At that time, Ferigolo [25–28] only briefly discussed the probable affinities of this specimen. Here, we present its full description and reevaluate the systematic position and affinities of *Clevosaurus brasiliensis* Bonaparte and Sues, 2006 [11]. We expect that this contribution will help to understand the taxonomic diversity of these Triassic/Jurassic sphenodontians.

## Geological Setting

**Fig 1:**
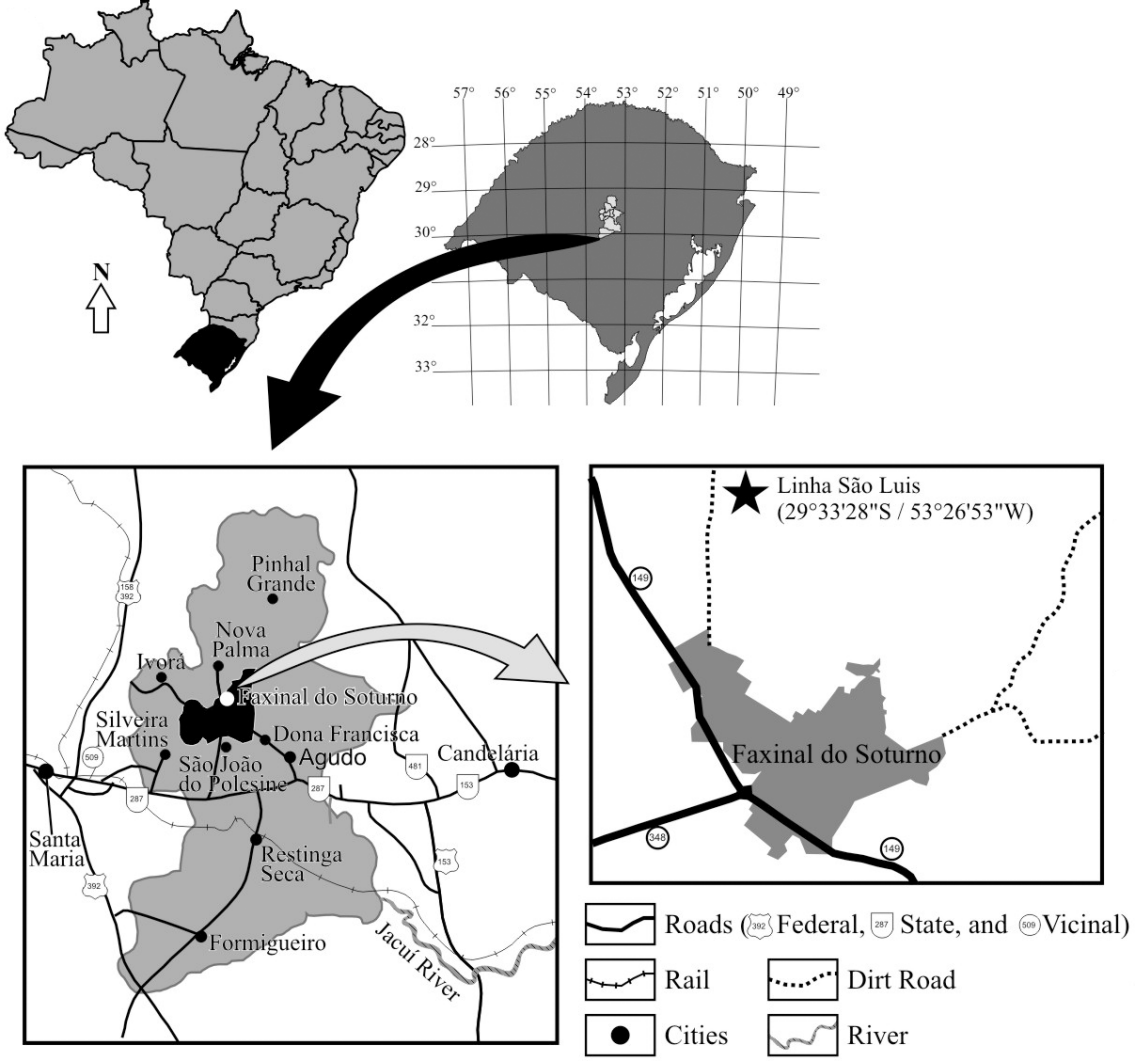
Location map. Sketch map of Rio Grande do Sul showing the outcrop area of the Santa Maria sequence (shaded) and approximate location of the type-locality (star) of *Clevosaurus brasiliensis* Bonaparte and Sues, 2006 [11].

The material was collected at Linha São Luiz locality (53°30’23"W, 29°34’08"S) (Fig 1), an abandoned quarry in the Faxinal do Soturno municipality, central region of Rio Grande do Sul, Brazil. The massive, fine-grained sandstone beds belong to the basal portion of the Late Triassic Caturrita Formation [29,30]. Sequence-stratigraphy schemes refer these strata to the upper part of ‘Sequence II’ of Faccini [31], or the highstand systems tract of the Santa Maria 2 Sequence [32]. The “Faxinal do Soturno Local Fauna” [33] shows a conspicuous small vertebrate assemblage, also including brasilodont [33–36] and tritheledontid cynodonts [33,34,37], and the procolophonid *Soturnia* [38]. Recent discoveries also include an undetermined non-rhynchocephalian lepidosaur [33]. Moreover, other sites referred to the Caturrita Formation have hielded the kannemeyerid dicynodont *Jachaleria* [39], the dinosauriform *Sacisaurus* [40], the basal saurischian *Guaibasaurus* [41–44], the “prosauropod” *Unaysaurus* [45], besides indeterminate sauropodomorph [46], phytosaur [47], and stereospondyl amphibian [48].

The fossils collected in this geological unit belong to the *Riograndia* Assemblage Zone [49–51]. Although a possible Early Jurassic age has been proposed [27], most authors agree on a Late Triassic age (possibly Norian) for the Caturrita Formation [41,43,46,50]. Records that indicate a Jurassic age includes a taphoflora composed of derived Bennettitales (*Williamsonia potyporanae*), conchostracans similar to the genus *Carapacestheria*, and footprints attributed to a large tridactyl theropod (*Eubrontes* isp). In sum, the “Faxinal do Soturno Local Fauna” may correspond to the latest Triassic or even the base of the Jurassic [52–54].

## Material and Methods

### Material

The sphenodontian clevosaur MCN-PV 2852 is housed at Museu de Ciências Naturais da Fundação Zoobotânica do Rio Grande do Sul (MCN/FZBRS), Porto Alegre, Brazil; it corresponds to incomplete, but well-preserved skull and mandible. Comparative material from the Caturrita Formation includes the cranial specimens of *Clevosaurus brasiliensis* (holotype UFRGS-PV 0748T; and referred specimens: UFRGS-PV 0613T, 1152T, 0735T, 0746T, 0753T, 0974T, 0972T, 0849T, 0754T, 0755T, 0752T, 0750T, 0749T, 0855T, 0738T, 0739T, 0737T, 0741T, 0758T, 1153T, 0745T0831T, and ULGV 9726). Data from the literature on *Brachyrhinodon taylori* and *Polysphenodon mulleri* were taken from Fraser and Benton [13], on *Clevosaurus hudsoni* from Fraser [20]; on *C*. *wangi*, *C*. *petilus* and *C*. *mcgilli* from Wu [23,55] and Jones [17]; on *C*. *bairdi* from Sues et al. [21]; and on *C*. *convallis* from Säila [18]. The osteological nomenclature and description mainly follows Fraser [20], Wu [23], Säila [18], and Jones [17]. The systematic arrangement mainly follows Apesteguía et al. [4].

#### Tree search and support measurements

The cladistic study was based on the most recent analysis in the literature [4]. The 74 employed characters are identical to those of the original analysis, unordered and equally weighted. Two characters (62 and 63) were scored as missing data (“?”) for all taxa in the original analysis. Here, these characters and scores are reproduced in order to keep the original enumeration. Regarding the OTUs (Operational Taxonomic Units) the Early Triassic lepidosauromorph *Sophineta cracoviensis* [56] was used to root the analysis intead of the basal Diapsida *Youngina capensis* (Late Permian of South Africa) [2,4,57]. The Squamata, represented only by the polychrotid extant lizard *Pristidactylus* in the original analysis, also includes *Eichstaettisarus* [58] from the Late Jurassic of Germany and Early Cretaceous of Spain and Italy, and a probably basal Gekkonomorpha [59] from the Early Cretaceous of Mongolia. The 30 original rhynchocephalians of the original analysis were maintained, with the addition of 11 new OTUs, codified based on the literature, namely: *Polysphenodon mulleri* [13–16,21,23,24,55,60], *Pelecymala robustus* [10–12], *Sphenotitan leyesi* [3], *Clevosaurus bairdi* [4–6,8], *C*. *wangi* [55], *C*. *petilus* and *C*. *mcgilli* [15,16,21,23,55,60], *C*. *convalis* [18], *C*. *latidens* (AUP 11192, formerly attributed to *Pelecymala*) [22,61], the South African *Clevosaurus* sp. (SAM K 7890) [62], and *Clevosaurus brasiliensis* [11]. The latter taxon was based on the holotype specimen (UFRGS-PV0748T, first hand examination) and the specimen of this present study (MCN-PV 2852), and their character states are scored in the single *Clevosaurus brasiliensis* used in the analysis. The present cladistic analysis was thus performed with 74 characters and 44 OTUs. The MPT heuristic search on TNT 1.1 [63,64] included 1000 replicates, with random addition sequences followed by tree-bisection-reconnection (TBR) branch swapping, retaining 10 trees per replicate and using collapse trees after the search. If some replications overflowed after a first round, traditional search was again employed, this time on trees from RAM. The verification of the original analysis [4] indicates the same results (22 most parsimonious trees of 218 steps), although *Gephyrosaurus* and *Pristidacylus* were found as sister taxa, and this clade as considered the sister group of other rhynchocephalians. Considering that *Gephyrosaurus* [65–67] is the most basal Rhynchocephalia, a constrain was incorporated into the cladistic analysis, enforcing the monophyly of that group. This latter analysis revealed the same number of most parsimonious trees (22), but with 219 steps. After all, the IterPCR protocol [68] was employed to identify unstable taxa and their possible phylogenetic position.

#### Fieldwork permit and repository information

All necessary permits were obtained for the described study, which complied with all relevant regulations. The field work and fossil collection was previously communicated to the Departamento Nacional de Produção Mineral ─ DNPM, based on ordinance n° 4.146 from March 4th, 1942. The specimens described in this work are housed in permanent collections: Coleção de Paleovertebrados do Museu de Ciências Naturais da Fundação Zoobotânica do Rio Grande do Sul (MCN-PV, Porto Alegre, Brazil); Coleção de Paleovertebrados da Universidade Federal do Rio Grande do Sul (UFRGS-PV, Porto Alegre, Brazil); Museu sobre a História Geológica do Rio Grande do Sul da Universidade do Vale do Rio dos Sinos (ULGV, São Leopoldo, Brazil).

## Results and Discussion

### Systematic paleontology

Lepidosauria Dumeril and Bibron, 1839 [69] (sensu Evans, 1984) [70]

Rhynchocephalia Günther, 1867 [71] (sensu Gauthier et al., 1988) [72]

Sphenodontia Williston, 1925 [73] (sensu Benton, 1985) [74]

Clevosauridae Bonaparte and Sues, 2006 [11]

Comments. Clevosauridae was first formalized by Bonaparte and Sues [11] to include “the last common ancestor of *Polysphenodon*, *Brachyrhinodon* and *Clevosaurus*, and all of its descendants”, based on previous phylogenetic studies of Wu [23] and Reynoso [14–16], which informally called that taxon as “clevosaurs group”. We define Clevosauridae as a branch-based group, composed by all taxa more closely related to *Clevosaurus* than to *Sphenodon*. Clevosaurids share an antorbital region forming ¼ of the skull length (reversed to between 1/3 to ¼ in *C*. *brasiliensis*, *C*. *wangi* and *C*. *petilus*), a narrow and elongated dorsal process of the jugal, and palatine teeth forming a single row plus one isolated tooth.

*Clevosaurus* Swinton, 1939 [75]

Commented diagnosis. According to some authors [3,5,13–15], *Clevosaurus* can be diagnosed based on the following features: suborbital fenestra bounded solely by the ectopterygoid and palatine; a dorsally expanded lateral process of the premaxilla; a high, steeply inclined coronoid process of the dentary; flanged teeth; a broad maxillary-jugal contact; maxilla excluded from the posterior margin of external naris. It is noteworthy that these characters are found in all *Clevosaurus*, but not restricted to the genus [17,18].

Type species. *Clevosaurus hudsoni* Swinton, 1939 [75]

Included species. *C*. *minor* Fraser, 1988 [20]; *C*. *latidens* Fraser, 1993 [22], *C*. *wangi*, *C*. *mcgilli* and *C*. *petilus* Wu, 1994 [23]; *C*. *bairdi* Sues et al., 1994 [21]; *C*. *convalis* Säila, 2005 [18]; and *C*. *brasiliensis* Bonaparte and Sues, 2006 [11]

*Clevosaurus brasiliensis* Bonaparte and Sues, 2006 [11]

1999 gen. et. sp. indet. Ferigolo 1997: 24–25. [25]

1999 gen. et. sp. indet. Ferigolo et al. 1999: 60–60. [26]

2000 gen. et. sp. indet. Ferigolo 2000: 236–245, Figs four to six [27]

2009 gen. et. sp. indet. Ferigolo 2009: 89–105, Figs five and six [28]

Holotype. UFRGS-PV 0748 T, an incomplete but well-preserved skull, with mandibles attached. The specimen is housed at Coleção de Paleovertebrados da Universidade Federal do Rio Grande do Sul, Porto Alegre, Brazil (UFRGS-PV).

Type horizon and locality. Linha São Luiz locality, Faxinal do Soturno Municipality, Caturrita Formation, Late Triassic, Late Norian-Rhaetian age [16].

Emended diagnosis. *Clevosaurus brasiliensis* is a clevosaurid sphenodontian distinct from other Clevosauridae by a posterior parietal edge slightly incurved inward, a parietal foramen at the same level of supratemporal fenestra, angle between anterior margin and the longitudinal axis of mandible less than 120º in lateral view, and absent or inconspicuous posteromedial flanges on the posterior maxillary teeth and anterolateral flanges on the mandibular teeth.

**Fig 2:**
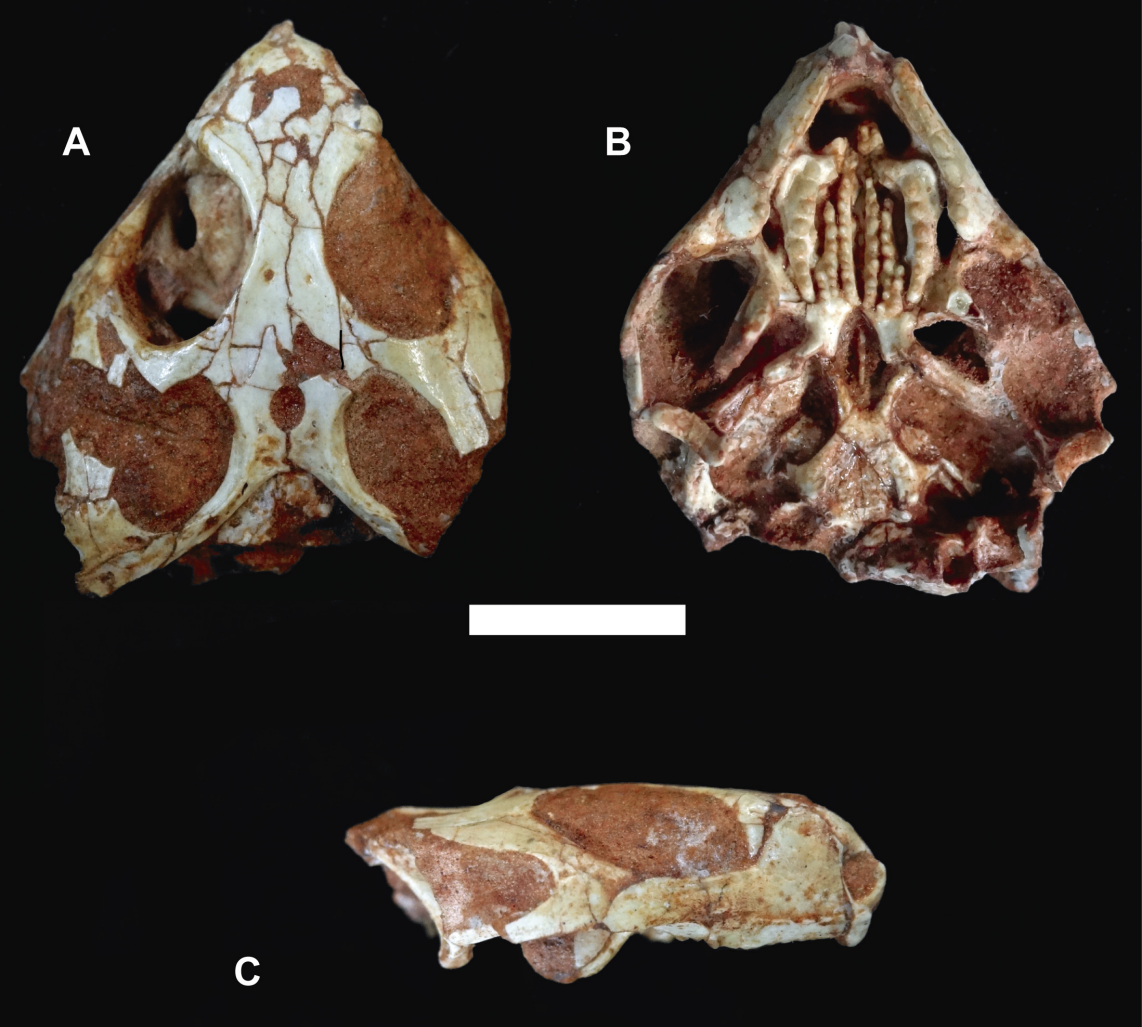
*Clevosaurus brasiliensis* Bonaparte and Sues, 2006 [11] MCN-PV 2852, skull. Photographs (A-C) in dorsal (A), palatal (B), and lateral (C) views. Scale bar equals 5 mm.

**Fig 3:**
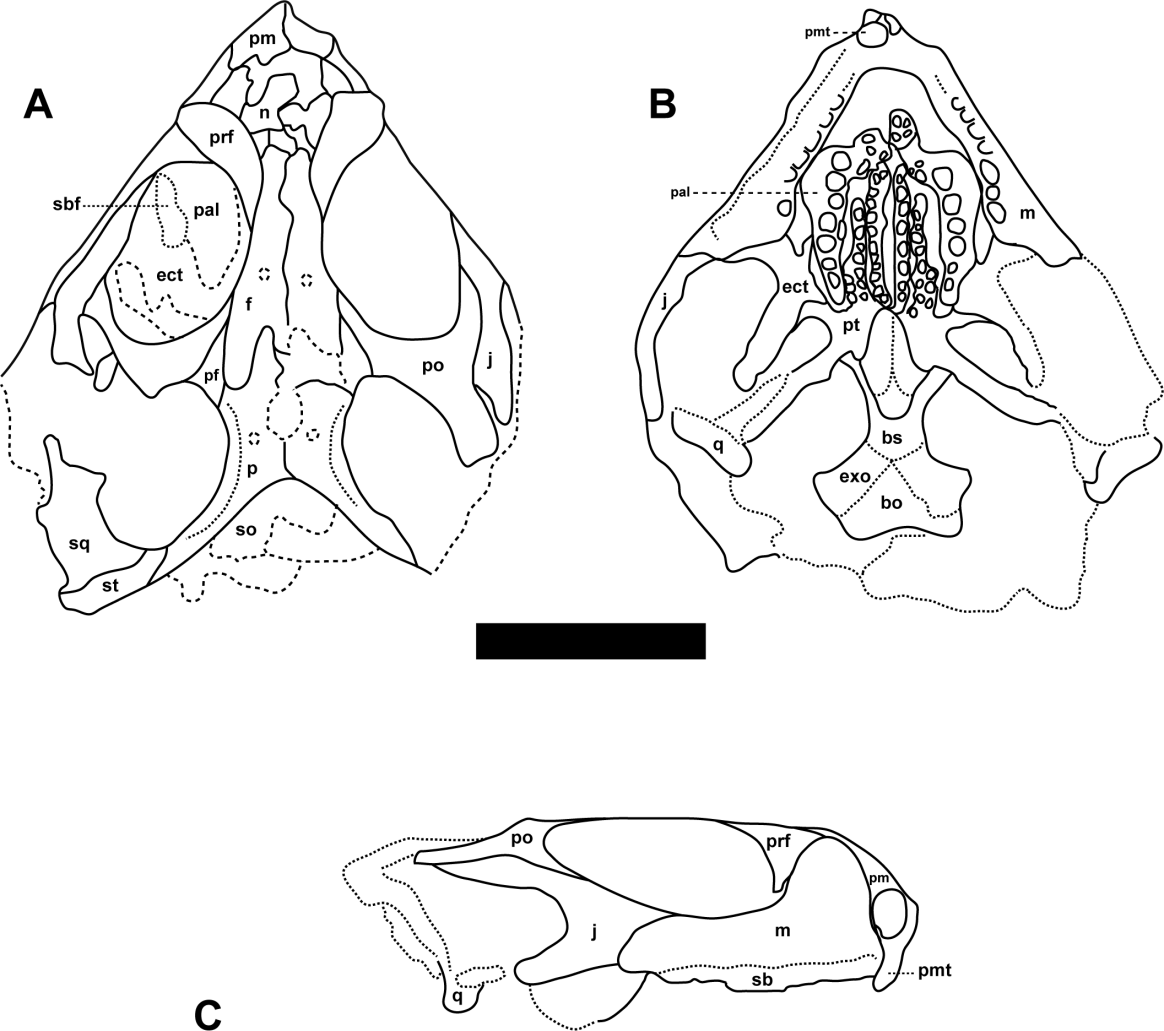
*Clevosaurus brasiliensis* Bonaparte and Sues, 2006 [11] MCN-PV 2852, skull. Schematic drawings (A-C) in dorsal (A), palatal (B), and lateral (C) views. Scale bar equals 5 mm. Abbreviations: bo, basiocciopital; bs, basisphenoid; ect, ectopterygoid; exoccipital; f, frontal; j, jugal; m, maxilla; n, nasal; p, parietal; pal, palatine; pf, postfrontal; pm, premaxilla; pmt, premaxilla tooth; po, postorbital; prf, prefrontal; pt, pterygoid; q, quadrate; sb, secondary bone; sbf, suborbital fenestra; so, supraoccipital; sq, squamosal; st, supratemporal.

**Fig 4:**
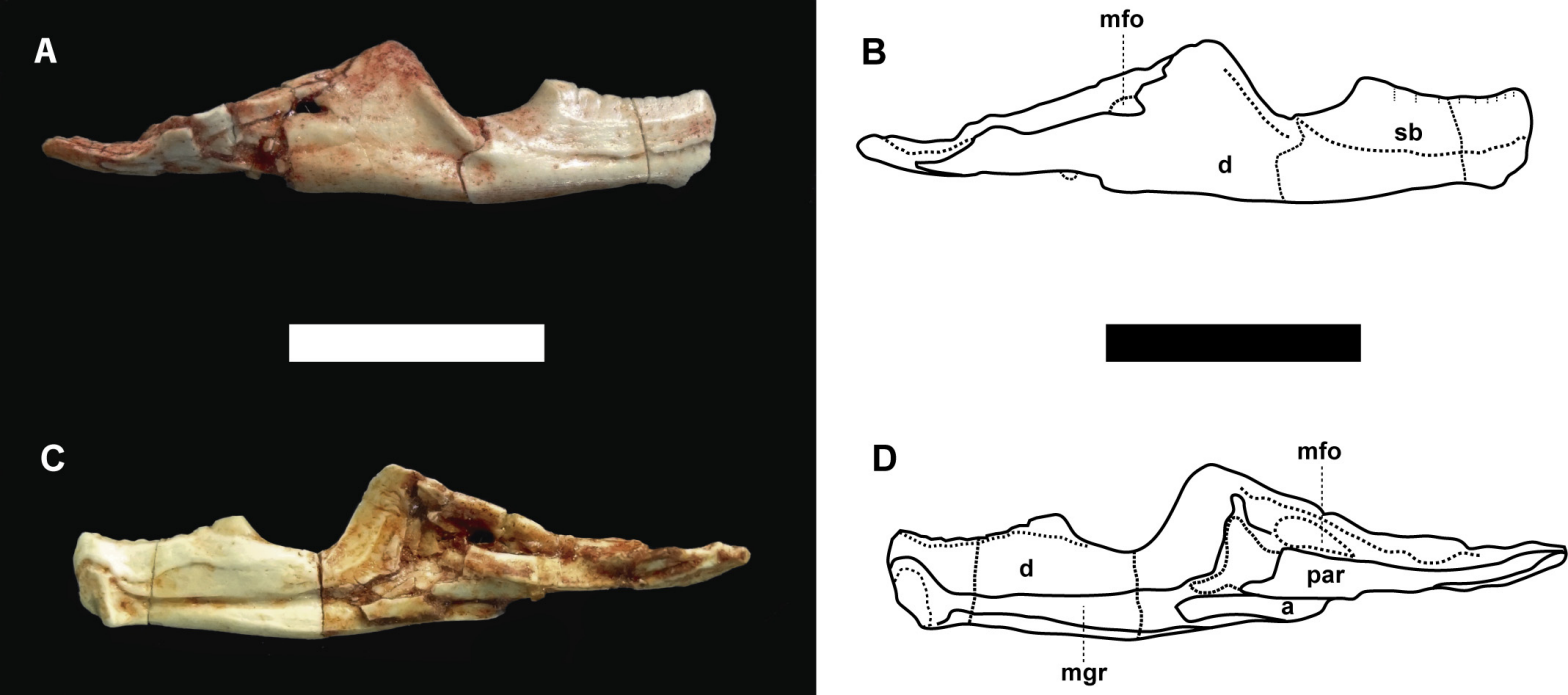
*Clevosaurus brasiliensis* Bonaparte and Sues, 2006 [11] MCN-PV 2852, right dentary. Photographs (A and C) and schematic drawings (B and D), in lateral (A-B) and lingual (C-D) views. Scale bar equals 5 mm. Abbreviations: a, angular; d, dentary; mfo, mandibular foramen; mgr, Meckel’s groove; par, prearticular; sb, sencondary bone.

Referred specimen. MCN-PV 2852, well-preserved skull with associated mandibles (Figs 2–4). The specimen is housed at the Coleção de Paleovertebrados do Museu de Ciências Naturais da Fundação Zoobotânica do Rio Grande do Sul, Porto Alegre, Brazil (MCN-PV).

Stratigraphic and geographic range. The material MCN-PV 2852 was recovered from the same type locality of the holotype of *C*. *brasiliensis* Bonaparte and Sues, 2006 [11], at Linha São Luiz locality, Faxinal do Soturno Municipality, Caturrita Formation, Late Triassic (Late Norian-Rhaetian age, *sensu* [50].

### Description of *Clevosaurus brasiliensis* based on MCN-PV 2852

#### General features of the skull

The skull of MCN-PV 2852 is nearly 25.5 mm long. It is slightly compressed on the left side, some bones are crushed (basioccipital, exoccipital), or very damaged (supraoccipital, prootic, opisthotic) (Figs 2 and 3). Other bones, as epipterygoid and stapes are missing. Few cervical vertebrae superpose posteriorly over the braincase. The lower jaw is almost complete and 26.8 mm long. The antorbital region (snout) of MCN-PV 2852 is relatively short, between ⅓ and ¼ of the total skull length, as in the holotype of *Clevosaurus brasiliensis*. The supratemporal fenestra is more than ¼ of the skull length, same pattern seen as in *C*. *wangi*, *C*. *petilus*, and *Polysphenodon* (¼ or less). The temporal region is broad as in most *Clevosaurus* species, except from the narrow temporal region of *C*. *hudsoni*, [23], and from *C*. *bairdi*, in which this region is incomplete.

#### Dermatocranium

Premaxillae and maxillae (Figs 2 and 3). Only the left premaxilla is complete. The anterodorsal and posterodorsal processes are thin and form the ventral, the posterior, and half of the anterior margins of the oval-shaped external naris. The anterodorsal process is slightly prominent, and does not contact between the nasal bones; whereas the posterodorsal process extends dorsally between nasal and maxilla. Both maxillae are well preserved. Anteriorly, the maxilla is excluded from the external naris by the posterodorsal process of premaxilla, a feature shared with all *Clevosaurus* [14,17,21,23], but also present in *Godavisaurus*, *Opisthias* and *Priosphenodon* [17,18]. The large dorsal process is deep and rounded, and its posterior border seems to be overlapping the anterior margin of the ventral process of the prefrontal, except at the anteroventral corner of the orbit. At this point, the broad contact between the maxilla and jugal (seen in all *Clevosaurs* species, but also in some other sphenodontians) [17,76] form the lateral margin of the small and dorsally open lacrimal foramen. The long posterior process, with a rounded caudal margin, forms most of the ventral edge of the orbit, overlapping the anterior process of jugal in all its extension. In ventromedial view, the maxilla seems to be included in the suborbital fenestra, which closes part of the anterior side wall of the suborbital fenestra. However, in dorsal view it is excluded, with the suborbital fenestra limited only by the palatine and ectopterygoid, without participation of maxilla (also observed in *Clevosaurus bairdi*, *C*. *hudsoni*, and *C*. *minor*) [20,21], and considered exclusive to *Clevosaurus* [17]. The palatine process is narrow and small, and contributes to form the posterior margin of the internal naris. There are at least five supralabial foramina (on the left side) just above the secondary bone or secondary dentine “lip” of the maxilla [20].

Nasals, prefrontals, and frontals (Figs 2 and 3). Despite the poor preservation of both nasals some important features can be noted. The nasal is fused to the frontal and broader at the level of the anterior margin of prefrontals, as in the holotype of *Clevosaurus brasiliensis*. It also forms the anterior half and the entire dorsal margin of the external naris.

The large semilunar prefrontal forms all anterodorsal margin of the orbit. In the intraorbital surface, the ventral process of the prefrontal contacts the palatine. The triangular and pointed frontal process overlaps the lateral margin of palatine, whereas the ventral process (although slightly disarticulated) is overlapped by the anterodorsal process of maxilla. The prefrontal contributes together with the jugal to form the medial margin of the lacrimal foramen. Only in *Clevosaurus hudsoni* the contact between the prefrontal and the jugal is not visible, although Sues et al. [21] based on isolated maxillae of *C*. *hudsoni* interpreted that the articular facets for the prefrontal and jugal could indicate that the infraorbital process of the jugal closely approaches the ventral portion of the prefrontal. Both frontals are complete, fused to nasals, but not fused with parietals.

The frontal contributes with the middle portion of the dorsal margin of the orbit, where it largely overlaps the postfrontal, almost touching laterally the postorbital. The posteromedial process is short and narrow. Right and left frontals are wedged between the anterior processes of parietals, whereas the posterolateral process is longer and wider, with a convex lateral margin covering the postfrontal. At the interorbital region there is a pair of small vascular foramina.

Postfrontal and postorbital (Figs 2 and 3). Only the left postfrontal is preserved and the exposed area is slightly wide at both extremities: anteriorly participating in the medial margin of the orbit, and posteriorly forming the anteromedial margin of the supratemporal fenestra. It is distinct from the holotype of *Clevosaurus brasiliensis* where it is broad with a large medial contact with the parietal and anteriorly participating in the most posterior margin of the orbit, as well as the anteromedial border of the supratemporal fenestra [11]. The postfrontal morphology resembles the condition observed in *Clevosaurus hudsoni*, with a shorter posterior process overlaping the anterolateral margin of the parietal [20].

Although incomplete, the postorbital has a Y-shape and contributes to form the margin of the orbit and the supratemporal fenestra. The postorbital has short medial and ventral processes, but possesses a long posterior projection. The medial process widely overlaps the postfrontal, and forms all the posterior margin of the orbit, as well as the anterolateral margin of the supratemporal fenestra. The ventral process forms more than half of the posterior margin of the orbit, and the posterior process (incomplete) possibly formed all the anterior half of the lateral margin of the supratemporal fenestra.

Jugal (Figs 2 and 3). The right jugal is almost complete. Although largely overlapped by the posterior process of the maxilla, medially it is a long anterior process that reaches the anterior edge of the orbit, participating with the prefrontal in the medial margin of the lacrimal foramen. According to Wu [23], the jugal of *C*. *petilus* is distitinct from other *Clevosaurus*, because this bone has an anterior process that forms only a small portion of the posteroventral margin of orbit. The long and backward oriented dorsal process, contacts anteriorly the ventral and dorsally the posterior process of the postorbital, and also meets anteriorly the squamosal, as observed in *Clevosaurus hudsoni*, *C*. *bairdi*, *Brachyrhinodon*, and *Polysphenodon* [17]. The jugal forms the ventral half of the posterior margin of the orbit, and almost one third of the dorsal margin of the lower temporal fenestra; its ventral margin is formed by the long posterior process (which in the holotype of *Clevosaurus brasiliensis* is fragmented). This process, although anterior and medially displaced, still contacts the quadrate complex, and it appears that the lower temporal bar is complete (as in *Clevosaurus hudsoni*, *C*. *minor*, and *C*. *bairdi*) [20,21]. According to Jones [17] this character is also found in basal rhynchocephalians, however, the difference between the condition seen in clevosaurs and basal forms could be due to the larger postorbital area, as well as in *Sphenodon*, where the postorbital area the jugal does not extend posteriorly to meet the squamosal.

Squamosal (Figs 2 and 3). Only part of the left squamosal was preserved. The entire posterior border of the bone contacts the supratemporal. The anterior process of the squamosal displays two articular facets: the lateral one for the jugal (dorsal process), and the medial one for the postorbital. The bone forms one third of the lateral margin of the supratemporal fenestra, as well as the posterior one third of the dorsal margin of the lower temporal fenestra. Only a small portion of the descending process was preserved, which is also dorsally displaced.

Parietals (Figs 2 and 3). The parietals are almost complete, and the interparietal suture displays an interdigitating pattern. The parietal region is wide, as in *Brachyrhinodon*, *Polysphenodon*, Chinese *Clevosaurus*, and the holotype of *Clevosaurus brasiliensis*, but distinct from *Clevosaurus hudsoni* and *C*. *bairdi*, where it is relatively narrower than the interorbital region. The condition found in MCN-PV 2852 and in the holotype of *Clevosaurus brasiliensis* resembles the parietal table morphology of *C*. *hudsoni*: the tendency to form a median ridge [20]. However, it is different from the condition seen in *Palaeopleurosaurus*, *Kallimodon*, *Sapheosaruus*, and *Sphenodon*, where a prominent parietal crest, or a “true” median ridge, is present [20,23]. In MCN-PV 2852 and the holotype of *Clevosaurus brasiliensis*, the anterior margin of the large and oval parietal foramen reaches the level of the anterior margin of the supratemporal fenestra. The anterior process is large, triangular, and wedged between the posteromedial/posterolateral processes of frontal. The very long posterior process of parietal contributes with more than half of the posterior margin of the skull, and tapers abruptly at the contact with supratemporal, but a discrete contact with the squamosal perhaps occurs only at the internal surface of these bones.

Supratemporal (Figs 2 and 3). Only the left supratemporal was preserved, lacking the posterolateral edge, and its anterior margin contacts the squamosal. Its medial margin tapers anteriorly to contact with the posterior process of parietal bone, where it seems not participate in the margin of supratemporal fenestra. The bone forms about one third of the posterior margin, and probably the posterior edge of the skull. Regarding the presence of this bone in *Clevosaurus*, this character has often been interpreted as being exclusive to this taxon [17]. However, several other derived sphenodontians display the supratemporal bone, including *Zapatadon* and in the hatchling *Sphenodon* skull [6,15–17,77].

Vomer (Figs 2 and 3). Only a small posterior portion of the left vomer was preserved. Anteriorly, the vomer contacts dorsally the anterior process of pterygoid, more laterally and posteriorly it contacts the anterior process of palatine, including its very delicate semilunar (ventral) process. The preserved part of the vomer participates in the middle portion of the medial margin of the internal naris. There are two vomerine rows of small conical teeth, a medial one with three, and a lateral one with two teeth. These small teeth are similar in size to that of the pterygoid teeth or even smaller.

Palatines (Figs 2 and 3). Both enlarged and slightly curved palatines are preserved. This bone in the holotype of Clevosaurus brasilensis is partially hidden by the lower jaws and for this reason a detailed description is not possible. However, MCN-PV 2852 displays all palatal region exposed. Anteriorly, a triangular vomerine process shows ventrally a very small semilunar projection to joint with the vomer. Posterior to this facet, the process forms with the palatine process of the maxilla the posterior margin of the internal naris, as well as a little part of the medial margin of this opening. The semilunar tooth-bearing portion has a very particular morphology. It is wider anteriorly, where there is a small, but deep medial rounded process, which together with the vomerine process recovers the most anterior and toothless part of the palatal shelf of the pterygoid bone. MCN-PV 2852 possesses a shorter contact between the pterygoid and palatine, resembling a vacuity between these two bones, whereas in the holotype of Clevosaurus brasiliensis the contact is broad.

The trapezoid and well developed maxillary process projects anterolaterally from the anterior half of the tooth-bearing portion, and it is as wide in this point. It displays a deep and wide wear groove for the occlusion of the lower jaw, laterally limited by a parallel rounded low crest [78]. Due to the large maxillary process, the tooth-row is much more medial in position than usual, as is the semilunar tooth-bearing portion as a whole, which posteriorly tapers to wedge between the pterygoid shelf and the flange of ectopterygoid. Its posterior half contacts laterally the palatine shelf of the ectopterygoid, between the posterior margin of the suborbital fenestra and the posterior contact of the palatine with the transverse flange of the pterygoid. The pterygoid shelf is almost semilunar and tapers both anterior and posterior portions: anteriorly wedged between the rounded medial process, and the medial tooth-row of the pterygoid; and posteriorly wedged between the lateral tooth row of the pterygoid and tooth row of the palatine. The palatine contributes to the margins of the suborbital fenestra: the anterior one is formed by the maxillary process, whereas the middle part of the tooth-bearing portion forms the anterior half of its medial margin. This fenestra, due to the large maxillary process of the palatine and the relationship of this bone with the ectopterygoid, is nearly fusiform-shaped and relatively short. Also due to the width of the maxillary process, the tooth row of the palatine is unusually medial, so that the posteriormost tooth is only a few millimeters more lateral than the anteriormost one.

The tooth row bears seven (right side) and six (left side) conical teeth: the first two are the largest ones and the other teeth reduce their size posteriorly, so that the last ones are similar in size to the largest pterygoid tooth. The first teeth have large wear facets. In the right pterygoid shelf, medial to the fourth tooth in the row, there is an isolated tooth, similar in size to those in the row. In the rounded medial projection of the tooth-bearing portion there are three very small conical teeth posteromedially directed. On the left side, there is another isolated tooth (completely worn-out) inside the wear groove, in a level just anterior to the first tooth in the row. Most sphenodontians has a single palatine tooth row, where the teeth are conical and unflanged, but they bear small posterolateral flanges in *Godavarisaurus* [76], while there are also small flanges on the anterior palatine teeth of *C*. *hudsoni* [18].

Pterygoids (Figs 2 and 3). Both pterygoids are almost complete. The central region is rectangular and barely longer than wide, bearing a median sulcus, which is the very evident interpterygoid groove. From the lateral portion of the central area projects ventrally a thick semilunar process, just anterior and ventrally to the basipterygoid joint. The central area and the semilunar process are continuous, projects laterally the rod-like transverse process, which anteriorly contacts the posterior corner of the tooth-bearing portion of palatine (more medially) and the posterior limit of the palatine shelf of ectopterygoid (more laterally). From the lateral limit of its contact with the ectopterygoid flange, the transverse process continues by a delicate blade (seen in oblique views), that contacts ventrally the dorsal border of the posterior process of ectopterygoid. Only the left side the proximal portion of the quadrate process was preserved. The semilunar socket for the basipterygoid process can be observed in the palatal view, between the posterior border of the central area and the basis of the quadrate process, just dorsal to the semilunar process.

The most conspicuous feature observed in the palatal shelf of the pterygoid is the presence of three tooth rows on the palatal shelf. The most lingual (medial) row bears 13 teeth at the right side, and 10 at the left side, all them of similar size. The intermediary row bears eight teeth of different size; and the very small buccal (lateral) row has five teeth, all of similar size. Although Bonaparte and Sues [11] have described the third row as an “extra pterygoid teeth”, our observations suggest the presence of the well-developed third pterygoid buccal tooth row, including seen in the complete specimens of *Clevosaurus brasiliensis* (ULGV 9726, UFRGS PV 0746 T, UFRGS PV 1152 T). It is noteworthy that extra pterygoid teeth rows are also seen in basal rhynchocephalian forms, such as *Gephyrosarus* and *Dyphydontosaurus* [65,79], while derived forms have no tooth row in the pterygoid shelf [6,17], although the opistodonthian *Sphenotitan* has four pterygoid tooth row [3]. The available information about the pterygoid tooth row in *Clevosaurus* shows that most of them have two pterygoid teeth rows parasagitally aligned with each other. As observed in some Chinese *Clevosaurus* specimens [17], whereas *Clevosaurus brasiliensis* displays another pterygoid tooth row aligned to the most posterior portion of the palatine, but not a posterior continuation of the palatal tooth row. According to Jones [17] the specimen attributed to *C*. *wangi* (IVPPV 82710) apparently has more than two pterygoid tooth row, but was not figured in the original article of Wu [23], resembling the condition observed in *C*. *brasiliensis*.

The very long interpterygoid groove extends between the two lingual rows until the level of the most posterior pterygoid teeth of the intermediary row. In other words, both sides of the palatal shelves of the lingual row are sutured each other forming a clear and evident groove. The interpterygoid groove is totally closed by the posterior facet of the lingual row of each side in MCN-PV 2852 and ULGV 9726, but its widely open in the holotype of *Clevosaurus brasiliensis* and another specimen (UFRGS PV 0746 T), which could be interpreted as an intraspecific variation trait.

Ectopterygoids (Figs 2 and 3). The right ectopterygoid is preserved. The anterolateral process is narrow and long forming the posterior margin of the suborbital fenestra, and posteriorly the ectopterygoid contacting laterally two-thirds of transverse process of the pterygoid. Medially, contacts by all its extension the posterior half of the tooth-bearing area of the palatine. The lateral process lacks its rod-shaped portion, and is reduced to its lateral extremity. From this point, there is an anterior thin and long ramus, projecting from the lateral limit of the flange and extending (as seen inside the orbit) alongside of the medial surface of the maxilla (lateral margin of the suborbital fenestra) to contact more anteriorly the delicate posterior extremity of the maxillary process of the palatine. The lateroposterior ramus is robust, and is directed towards the posteromedial corner (posterior to the last tooth) surface of maxilla, a position related to the markedly inset position of the posterior extremity of the tooth-bearing portion. The crescent-shaped, robust and long posterior process is markedly concave laterally, having the ventral and distal end borders rounded.

#### Neurocranium

Parabasisphenoid and basioccipital (Figs 2 and 3). The limits between parasphenoid and basisphenoid can be partially distinguished. The fusion of the hexagonal basisphenoid with the parasphenoid is clearly at the posterior margin of internal carotid artery foramina, whose margins are formed by parasphenoid (anterior and medially), and basisphenoid (posteriorly), and the medial basis of the basipterygoid process (laterally). A thin and long cultriform process broadens posteriorly by means of the lateral (right/left) narrow alae; between which, at the midline, there is a relatively thick and rounded (ventral) crest. The long and deep basipterygoid process projects from the anterolateral border of the basisphenoid, laterally to the sulcus for the carotid artery. It is ventrally directed to articulate with the pterygoid, just medial to the quadrate process. Its ball-and-socket joint, exposed in ventral view, seems to have been a movable joint. Between the basisphenoid and the exoccipitals it is possible to see a V-shaped line, marking the previous suture; a line that is open at the apex, by a delicate transverse sulcus, evincing the previous contact between the basisphenoid and basioccipital bones. From the contact-line with the basisphenoid, the exoccipital widens posterolaterally, but the condyle cannot be distinguished. Between the exoccipitals there is a depression, anteriorly convex, perhaps pointing out the limit of these bones with the basioccipital. Although badly preserved, it is possible to see a laterodorsal projection from the basioccipital. Other portions of basioccipital and exoccipitals, supraoccipital and prootic/opisthotic are too crushed.

#### Splanchnocranium

Quadrate (Figs 2 and 3). The quadrate complex is incomplete in both sides, and the quadratojugal could not be distinguished from the quadrate, possibly being fused to each other. Due to the skull compression, at the right side there is only a partial contact between the quadrate and the pterygoid due to the fact that the respective processes are anteromedially displaced. At left side, the quadrate articular facet for the pterygoid can be observed, on account of the fact that the posterior portion of the jugal is missing. The facet of the condyle is wide but relatively short, markedly concave ventrally from side to side, with a convex anterior and a slightly concave posterior margin. The medial extremity of the condyle projects strongly ventrally, whereas its lateral extremity is almost horizontally flat. At the right side it is possible to observe the medial half of the conch-like basin, which is part of the quadratojugal foramen, and the column of the quadrate, but the head and the tympanic crest are missing.

Mandible (Fig 4). The tooth-bearing portion of the lower jaws are short so that the postcoronoid portion is longer than the precoronoid one. The coronoid process is high, triangular, and well-developed. In occlusion, the long retroarticular process surpasses the posterior limit of the skull, and is formed dorsally by the articular and ventrally by the angular, which tapers anteriorly almost to the mandibular incisure. There are eight small mentalia foramina at the shallow lateral longitudinal sulcus. From the apex of the coronoid process, a well-marked crest extends posteriorly until the posterior limit of the retroarticular process; whereas ventrally it is prolonged by a rounded and strong crest that together with the prominent ventral margin of the dentary forms a shallow V-shaped depression. Laterally, at the dentary-surangular suture, there is a large, horizontal, oval mandibular foramen.

The posterior process of the dentary is high and long and almost reaching the glenoid level. The symphyseal area is oval, almost vertical, having three portions: a dorsal semilunar gap, separated from an oval symphysis by a shallow Meckel’s groove, ventrally to which there is another smaller gap (as in *Sphenodon*) that is just above a small symphyseal process. As seen in all *Clevosaurus*, the symphyseal spur *sensu* [2], is reduced and roudend. The Meckel’s groove runs from the symphyseal area until the posterior limit of the basis of the coronoid process. Also departing from this sulcus, but dorsal to it, a very thin groove ends at the anterior limit of the mandibular incisure. The surangular is long and rod-like where it forms the dorsal margin of the adductor fossa, its ventral margin being formed by a rod-like prearticular which is slightly larger posteriorly. The articular facet (glenoid fossa or cavity) for the quadrate, located posterior to the surangular-articular contact, is relatively complex, with lateral and medial portions. The articular facet is a few millimeters anterior to the medial one, being slightly larger, concave and nearly oval in transverse section. It faces dorsally, and its posterior limit lies in a low transverse crest (there is no anterior limit). The medial portion, slightly smaller and posterior to the lateral one, is tilted towards the midline, is also nearly oval but its concavity is oriented longitudinally. Between both portions there is a low and rounded longitudinal crest. Posterior to the articular facet, a low crest runs over, ending at the well-developed posterior extremity of the retroarticular process. The angular contacts the articular near the lateral limit of the articular facet, and extends to the posterior margin of the retroarticular process.

#### Dentition

Teeth (Figs 2–4). The premaxillary tooth is ~2.0mm high and 1.3mm long at the base. As seen in the holotype of *Clevosaurus brasiliensis* (UFRGS PV 0748 T), each premaxilla of the MCN-PV 2852 only has a single tusk-like ‘incisor’ tooth, as in *Clevosaurus bairdi* [11,21] and Chinese *Clevosaurus* [17,23]. On the other hand, *C*. *hudsoni* and *C*. *convalis* have 3–4 premaxillary teeth [18]. The maxilla shows seven to eight teeth of the hatchling dentition, all conical, small, with an alternating size. Hatchling teeth are followed by other three teeth of the additional series: the first one is buco-lingually compressed, following the “clevosaurs tendency” [80] being larger than the other two, which are much smaller and conical. There are no anterolateral flanges on the maxillary teeth of MCN-PV 2852, and is also shared by *Brachyrhynodon* and all others *Clevosaurus*. However, the presence of large posteromedial flanges is seen in the additional teeth of the maxilla in all *Clevosaurus* [76], including *C*. *hudsoni* and *C*. *convalis* [18], but are not seen in MCN-PV 2852 and the holotype of *C*. *brasiliensis*. According to Bonaparte and Sues [11] the maxillary dentition of the holotype of *Clevosaurus brasiliensis* has two large flanged teeth, however, these features cannot be seen in more detail due to the close position of the mandibles with the skull. Probably, MCN-PV 2852 and UFRGS PV 0748 T (holotype of *Clevosaurus brasiliensis*) are old adults, evidenced by the high degree of wear [78]. Probably due to this reason they do not show any evidence of flanges on the dentary, maxillary or palatine dentition, although there is a subtle parallel vertical sulcus in the lower jaw, which could be related to a precision shear bite (orthal shear), *contra* [27].

The dentary possesses a slightly larger first tooth, this is followed by nine small, alternating teeth, eight of them corresponding to the hatchling dentition. The last tooth is larger than the others, corresponding to an additional tooth. According to Säila [18], there are enlarged anterolateral flanges on the dentary of *Clevosaurus* [20], but not observed in MCN-PV 2852 and holotype of *C*. *brasiliensis* (UFRGS PV 0748 T).

Maxillary and dentary heavy tooth wear indicates an adult stage. Hatchling dentition and additional series of maxilla shows important wear at the lingual side, due to occlusion with the lower jaw. In the palatine bone, wear is at the labial side of the first, second and third teeth at the right side; and at the first and second teeth at the left side. A conspicuous wear is also present inside the wear groove for the lower jaw; where at the left side an isolated tooth is now completely worn-out. The lower jaw teeth shows important wear at the labial side (maxillary occlusion), at the lingual side (palatine tooth row occlusion), and occlusal surface (wear groove of the palatine). Wear is also present at the labial side of the dentary, made by the maxillary teeth, and possibly at the lingual side of the maxilla, made by dentary teeth, just below the basis of the teeth.

### Phylogenetic Relationships

According to Wu [23] and Reynoso [14] the “clevosaurs group” comprises Brachyrhinodon, Polysphenodon, and Clevosaurus, although other authors did not found such support for this clade [13,76,81]. Previous works have suggested that the “clevosaurs” are more derived than pleurosaurs [14,16,23], although more recent studies with different, but not necessarily more comprehensive, taxon sampling, suggest the opposite [17,21,22,82].

Regarding the monophyly of the “clevosaurs group”, Bonaparte and Sues [11] defined the family Clevosauridae, but did not perform a cladistic analysis. Arantes et al. [12] ascribed rhynchocephalian post-cranial materials from the same locality to *Clevosaurus brasiliensis* and, based on the Bonaparte and Sues [11], conducted a phylogenetic analysis that corroborated the monophyly of Clevosauridae. The group was regarded as “basal forms” among sphenodontians. The data matrix of [12] is mainly based on Apesteguía and Novas [6], which lack several *Clevosaurus* species. The present phylogenetic analysis includes most (seven) species of *Clevosaurus* (except for *C*. *minor*, based on uninformative material) [20], two other taxa of the “clevosaurs group” (*Brachyrhinodon* and *Polysphenodon*), and the possible *Clevosaurus* from South Africa (SAM K-7890) [62].

**Fig 5:**
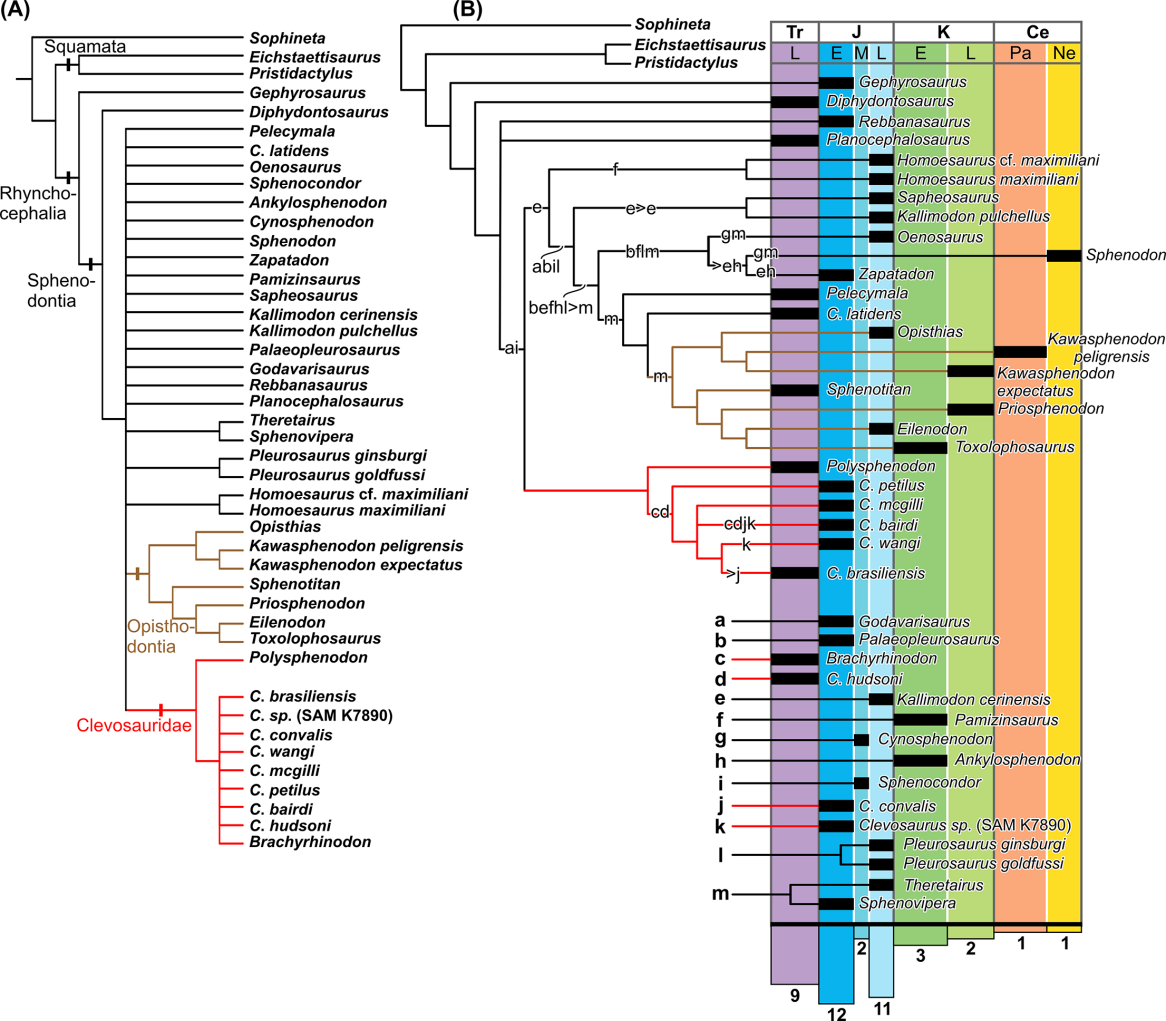
Results of cladistic analysis, recovered 14000 most parsimonius tress with 255 steps. A, Strict Consensus Tree. B, Tree obtained by iterPCR protocol (a-m indicate the unstable taxa pruned from cladogram and their possible positions).

The results reveal 1400 most parsimonious trees, with 255 steps. The strict consensus (Fig 5) shows *Diphydontosaurus* as most basal Sphenodontia. The others rhynchocephalians form a polytomy with only few clades revealed. Opisthodontia is recovered and better resolved than in the original analysis, probably because of the inclusion of *Sphenotitan*. This clade is composed by two groups: Eilenodontinae (*Sphenotitan* as most basal, with *Priosphenodon* as sister group of a clade that includes *Toxolophosaurus* and *Eilenodon*), and a clade composed by *Opisthias* and the two species of *Kawasphenodon*. In addition, sister group relationships are found between *Theretairus* and *Sphenovipera*; *Pleurosaurus ginsburgi* and *P*. *goldfussi*; and *Homeosaurus* cf. *maximiliani* and *Homeosaurus maximiliani*.

The Clevosauridae also is recovered as monophyletic, including *Polysphenodon*, *Brachyrhinodon* and *Clevosaurus*. Three characters support this clade: antorbital region forming ¼ of the skull length (character 1; reversed to a length between 1/3 and ¼ of that of te skull in *C*. *brasiliensis*, *C*. *wangi* and *C*. *petilus*, convergent with *Sphenotitan* and *Oenesaurus*); dorsal process of the jugal narrow and elongate (character 10; convergent with *Priosphenodon*, *Sphenodon*, and *Ankylosphenodon*); and palatine teeth forming a single row plus one isolated tooth (character 52; convergent with *Sphenotitan* and *Homoeosaurus* cf. *maxiliani*). *Polysphenodon* is the basalmost member of the group, with the other clevosaurids (*Clevosaurus* species and *Brachyrhinodon*) included in a polytomy. This clade is supported by two unambiguous synapomorphies: supratemporal fenestra 75% of the length or longer than orbit (character 3; convergent to *Sphenodon*, *Sapheosaurus*, *Kallimodon*, *Paleopleurosaurus*, *Pleurosaurus*, *Sphenotitan* and *Priosphenodon*) and supratemporal fenestra more than ¼ of the skull length (character 4; convergent with *Ankylosphenodon*, *Sapheosaurus*, *Kallimodon*, *Paleopleurosaurus*, *Homeosaurus* cf. *maxilmiliani*, and *Sphenotitan*).

The interPCR protocol [68] identified *Brachyrhinodon*, *C*. *hudsoni*, *C*. *convalis*, and *Clevosaurus* sp. (SAM-K7890) as unstable taxa within Clevosauridae (Fig 5). *Brachyrhinodon* could be the sister group of the clade including all *Clevosaurus* species, or closer to *C*. *bairdi*. Likewise, *C*. *hudsoni* could also have these two positions. Other *Clevosaurus* could also be closer to *C*. *bairdi*: *Clevosaurus* sp. (SAMP K-7890) and *C*. *convalis*. The iterPCR protocol also indicates that the South African *Clevosaurus* is not closer to *C*. *brasiliensis*, contradicting the expectation of a Gondwanan component in the evolution of *Clevosaurus*. This specimen could be closer to *C*. *bairdi* or to *C*. *wangi*. The possible position of *C*. *convalis* is closer to *C*. *bairdi*, or as sister group of *C*. *brasiliensis*.

In all trees of the analysis, *C*. *latidens* (AUP 11192) is not close to other *Clevosaurus*, and a position closer to Opistodontia, as proposed by Fraser [61] and Martínez et al. [3], was revealed by the iterPCR protocol. Among the Chinese *Clevosaurus* (*C*. *wangi*, *C*. *mcgilli*, and *C*. *petilus*), the iterPCR analysis indicates that they could correspond to distinct taxonomic entities. An elongate central region of the pterygoid (character 25) is only observed in *C*. *wangi* [23] among Clevosauridae. *C*. *wangi* also has a small mandibular foramen (character 37), shared with SAM K-7890 and *C*. *bairdi*, distinct from large foramen of *C*. *petilus* and *C*. *mcgilli*. The posterovental process of the jugal is well-developed (character 21) in most clevosaurids, except for *C*. *petilus* [23]. An antorbital region ranging between 1/3 and ¼ of the skull length (character 1) is shared only by *C*. *petilus* and *C*. *wangi*. Among clevosaurids, a posterior end of the palatine that widens posteriorly (character 22) is only observed in *C*. *petilus*, as well as the quadrate-quadratojugal foramen restricted to quadrate bone (character 28). Accordingly, although Jones [17] questioned the taxonomic validity of the Chinese *Clevosaurus* described by Wu [23], due to the poor preservation of the specimens, each of them have an unique condition among clevosaurids and share derived conditions with other *Clevosaurus*, indicating that they are possibly distinct and identifiable taxonomic entities.

## Conclusions

In this paper we report a new specimen of the clevosaurid sphenodontian *Clevosaurus brasiliensis* Bonaparte and Sues [11] from the Caturrita Formation, Late Triassic of Southern Brazil. The full comprehensive and detailed description of the specimen MCN-PV 2852 shortly studied by Ferigolo [25–28], allowed a better understanding of the skull morphology of *C*. *brasiliensis* compared to other *Clevosaurus*. The phylogenetic analysis, even with low resolution, reveals a monophyletic Clevosauridae, supported by the three synapomorphies: antorbital region one quarter or less of the skull length; narrow and elongate dorsal process of the jugal; and a single row of palatine teeth, plus one isolated tooth. The five exclusive characteristics present in *C*. *brasiliensis* among Clevosauridae, supports its taxonomic uniqueness: posterior edge of parietal slightly incurved inward (character 18); parietal foramen at the same level of the supratemporal fenestra (character 19); angle between the anterior margin and the longitudinal axis of the mandible less than 120° in lateral view (character 35), absent or inconspicuous posteromedial flanges on the posterior maxillary teeth (character 50); and absent of anterolateral flanges on the mandibular teeth (character 56). Analysing the cladistics results, it is evident the necessity of deeper morphological and cladistics studies to elucidate Rhynchocephalia (including *Clevosaurus*) evolution.

Together with the recent record of a basal sphenodontian in the Middle Triassic of Germany [83] and a basal opisthodontian in the Late Triassic of northern Argentina [3], the presence of *Clevosaurus* in the Late Triassic of Brazil suggests a great diversity of sphenodontians in the early Mesozoic, as shown by several other sphenodontians from the Late Triassic of Brazil still to be studied [84,85]. Additionally, the “Faxinal do Soturno Local Fauna” also includes a non-rhynchocephalian Lepidosauria, *Cargninia enigmatica* [33], as well as a questionable record of Lepidosauriformes [86]. Most fossils found in this locality are small tetrapods except for the basal saurischian *Guaibasaurus* [41–44]. According to Bonaparte et al. [33] the most significant contribution of the “Faxinal do Soturno Local Fauna” is to provide comprehensive information on the presence of very small Late Triassic tetrapods, not recorded in other coeval faunas from Argentina, Africa, India, and Australia. In this way, the “Faxinal do Soturno Local Fauna” shows that, as with medium and large sized tetrapods, there was a varied cast of small tetrapods of diverse phylogenetic significance [33].

## Supporting Information

S1 Information Matrix used in Phylogenetic Analysis. (PDF)

### Note Added in Proof

After the submission and, consequently, the acceptance of this manuscript, Klein et al. (2015) described a new species of *Clevosaurus*, *C*. *sectumsemper*, from the Rhaetian (Late Triassic) fissure fill in Lower Carboniferous Limestone, Fissure 1 in Woodleaze Quarry, South Gloucestershire, UK. Following these authors, *C*. *sectumsemper* is distinguished from other species of the genus by five characters: (1) the additional teeth bases on the dentary are more ventrally positioned with the final tooth base close to the upper edge of the Meckelian groove; (2) the pronounced antero-lateral flanges do not overlap or barely overlap the posterior flanges of the preceding tooth on the dentary; (3) the premaxillae each have two or three teeth, compared to the three or four of *C*. *hudsoni*; (4) a premaxillary chisel structure is not observed in any specimen; and (5) all the bones are small, no more than 80% the size of those in *C*. *hudsoni*. Although comparative analyses with other species of *Clevosaurus* were performed, is not clear if *C*. *sectumsemper* is a distinct taxon from *C*. *hudsoni* (Fraser, 1988) or *C*. *convalis* (Saila, 2005). Perhaps the inclusion of *C*. *sectumsempes* in the future cladistics analyses may resolve these issues on the phylogenetic affinities of this *Clevosaurus* taxon.

Klein CG, Whiteside DI, de Lucas VS, Viegas PA., Benton MJ. A distinctive Late Triassic microvertebrate fissure fauna and a new species of *Clevosaurus* (Lepidosauria: Rhynchocephalia) from Woodleaze Quarry, Gloucestershire, UK. Proc Geol Assoc. The Geologists’ Association. 2015; 126: 402–416. doi:10.1016/j.pgeola.2015.05.003
