# Supplementary material for: New Data on the Clevosaurus (Sphenodontia: Clevosauridae) from the Upper Triassic of Southern Brazil
Source: PLoS One. 2015 Sep 10;10(9):e0137523. doi: 10.1371/journal.pone.0137523 (PMC4565693; doi:10.1371/journal.pone.0137523)
Supplement: S1 Information — (PDF) [file pone.0137523.s001.pdf]

S1 Information: Matrix used in the Cladistic Analysis

xread

74 47

Youngina

00000000000000000002000000000?0000?0?00000002000000000000?10???00010??0000

Sophineta

00010000?0??00000?0?0???00??????00?0?????00??????????????????????????0000

Eichstaettisaurus

10110000010?011000000??000???00?0?????????????????????????????????????000?

Pristidactylus

11111000011001100001?0001000?000?00?0000000002000000???200?111??00010??0001

Gephyrosaurus

0000000000000110000120000000010100?000001000200000000000?102??00010??0001

Diphydontosaurus

1000000010000100000120100000010100001000111120010000000000121???000001?0000

Planocephalosaurus

00000000100001100101202000010101110000101212?0[0 1]20100001101211??1[1
2]00?1?0010

Rebbanasaurus

???????01??0???????????????????0?3102?????212200201001??101?????[0
1]11000100??

Godavarisaurus

??????1????????????????????????0110[0 2]?????212200[1 2]01021??100?????[0
1]10010100??

Homoeosaurus_maximiliani

1[0 1]0000011000?0000[1 2]11??2001???10111101?11121201[0
1]21202??2111222??1??????000?

Homoeosaurus_cf_maximiliani

11[0 1][0 1]??????0??00?0?11?02??1?????[0 1]1???1?11121[2 3]01[0 1]??20[1
2]??2??????2??1??????????

Palaeopleurosaurus

0111000010001011120020211011010011?1?1[0
1]1212011211020021012 10??022????0001

Pleurosaurus_goldfussi

0110?0001100?0011210002[1
2]101001020110??00121200120002102110?20??022????0000

Pleurosaurus_ginsburgi

0110?0001?00?001????0?????????0?0110?????21[2
3]0012000??0?110120??022????0000

Brachyrhinodon

211111011100?000000120120?????0011101?1012120112110100 1?????1??1????????21

Clevosaurus_hudsoni

2111111111010001000120210100000011101110121201121201001101211??112??0?0021

Kallimodon_pulchellus

01110?011?00?0011211112001???10[0
1]11021?10121201121202??2001221??122????00??

Kallimodon_cerinensis

??????????????????1??2010?????[0 1]110?1?[0 1]?1212??[0
1]21202002000221??122????00??

Sapheosaurus

111100?1?0???0011211211001????0[0 1]11021?10[0
1]???01?21?0???2???221??122?????????

Pamizinsaurus

??????1???0????????????211??0?0?[1 2]11?1?11?2120?[0
1]2?102???101?????12???1?????

Zapatadon

10?01??01??1??1?1?1?102210101[0 1]1?1112??1??2120?[0
1]??102??2????????12?????????0

Sphenodon

111110011100100111112122111011121112111112 1211[0 1]21102112100012??[1
2]2210000011

Cynosphenodon

?????????????????????????????1?2112??1??2121112110????100?????11210010011

Opisthias

????????????????????????????????3111??[0
1]??212010??20????112?????122????10??

Toxolophosaurus

??????????????????????????????1?31111?0??213012????????112?????222????01??

Eilenodon

??????????1???????????????????12311112022213012?2?21210?112?????222????0122

Priosphenodon

0110011111111001111101220100011231111202221301221212103112[1
2]2??222????0032

Ankylosphenodon

???1?????1???????????????????1?111?1?111?1?01????0????0?0221??121????0???

Sphenocondor

????????????????????????????????1?1?1?1?1212200????????0?0?????0[1
2]?010100??

Sphenovipera

??????????????????????????????1?21021???1212211????????112??????1[1
2]211??10??

Theretairus

??????????????????????????????1?210??????21221?????????1???????12?10??00??

Kawasphenodon_expectatus

??????????????????????????????1??????????212?10?????1??112??????[1
2]??????02??

Kawasphenodon_peligrensis

??????????????????????????????1??????????21[2
3]?10????????112????????????02??

Oenosaurus

20???0?1??0?10111????02201????11[2 3]?12111112??0?[0
1]????2?02000?????2?2????00?1

Polysphenodon

2000???11100?0000001?00??1????01???????01211?10211?1??1?????1????????????1?

Clevosaurus_bairdi

211111111101?001000120??010?00011??00110?21?010212?1??11?02????1?2??0?0021

Clevosaurus_petilus

111111111100?0?00001102101000001????1?10121??102?2?11011?0??????????????2?

Clevosaurus_mcgilli

20111111110100?00001212101010001?1?01?101212?10212?11011?0????????????002?

Clevosaurus_wangi

1?111111110?00?00001202111??0001????011?1212?10??201?001?0????????????002?

Clevosaurus_convalis

?????1???????????????0??????????1110??1??2120111?20??0?100?????1?2??0?00??

Clevosaurus_latidens

??????1???????????????????????1?3110?????212??2??1??????0???????221???????

Sphenotitan

21110111???11?11111121220?1001123111020012130122121110011??????1221002??0?

Pelecymala

?????001??????????????????????1?1????????212?12??10????10???????221?????0?

Clevosaurus_sp_SAMk

?????11??101?0?????12?2??????0??1????0???1?1????21??1??????????????????????

Clevosaurus_nrasiliensis_MCN

10111111110100000111202101??000111001110121201121001000000?????1?2??0?0021

Clevosaurus_brasiliensis_UFRGS

1011111011010000011??0210????00111?0??10121201121??1000??0?????1?2??0?0021

Clevosaurus_brasiliensis_unique

1011111[0
1]110100000111202101??000111001110121201121001000000?????1?2??0?0?21

;

proc /;
